# Supplementary material for: Genetic architecture of heart failure with preserved versus reduced ejection fraction
Source: Nat Commun. 2022 Dec 14;13:7753. doi: 10.1038/s41467-022-35323-0 (PMC9751124; doi:10.1038/s41467-022-35323-0)
Supplement: Supplementary file 3 — Description of Additional Supplementary Files [file 41467_2022_35323_MOESM3_ESM.pdf]

Description of Additional Supplementary Files

File Name: Supplementary Data 1
Description: Characteristics of non-HF controls and HF cases including clinical subtypes in the MVP cohort of non-Hispanic European (EUR) and non-Hispanic African Americans (AFR).

File Name: Supplementary Data 2
Description: Characteristics of non-HF controls and HF cases in the UK Biobank cohort of European ancestry.

File Name: Supplementary Data 3
Description: Summary statistics of genome-wide significant SNPs from the meta-analysis of unclassified heart failure. Meta-analysis was performed using inverse-variance weighted fixed-effects model. All tests were two-sided without adjustment for multiple testing.

File Name: Supplementary Data 4
Description: Summary of 20 genome-wide significant loci and sentinel variants associated with unclassified heart failure. Meta-analysis was performed using inverse-variance weighted fixed-effects model. All tests were two-sided without adjustment for multiple testing.

File Name: Supplementary Data 5
Description: Replication of previous reported variants associated with composite heart failure or dilated cardiomyopathy. All tests were two-sided without adjustment for multiple testing.

File Name: Supplementary Data 6
Description: Summary of genome-wide significant loci and sentinel variants associated with HFrEF or HFpEF. All tests were two-sided without adjustment for multiple testing.

File Name: Supplementary Data 7
Description: Associations with unclassified HF, HFrEF and HFpEF in autosomal genes related to cardiomyopathy. All tests were two-sided without adjustment for multiple testing.

File Name: Supplementary Data 8
Description: Genetic associations between HFrEF- and HFpEF-associated sentinel variants and heart failure risk factors. All tests were two-sided without adjustment for multiple testing.

File Name: Supplementary Data 9
Description: Genetic associations with ~2,400 traits tested in the UK Biobank searched in the PheWeb browser (listed as "-" if no associations with p<E-6 identified).

File Name: Supplementary Data 10
Description: Summary of Mendelian Randomization analyses between heart failure risk factors and unclassified heart failure, HFrEF and HFpEF using three methods. All tests were two-sided without adjustment for multiple testing.

File Name: Supplementary Data 11
Description: Independent secondary signals within the genome-wide significant loci identified by conditional analysis. All tests were two-sided without adjustment for multiple testing.

File Name: Supplementary Data 12
Description: Candidate causal variants from credible set analysis for all genome-wide significant loci. All tests were two-sided without adjustment for multiple testing.

File Name: Supplementary Data 13A
Description: SIFT prediction scores for non-synonymous substitution of amino acid.

File Name: Supplementary Data 13B
Description: PolyPhen prediction scores for non-synonymous substitution of amino acid.

File Name: Supplementary Data 14
Description: Expression quantitative trait loci (eQTLs) for the genetic variants associated with unclassified HF, HFrEF and HFpEF.

File Name: Supplementary Data 15
Description: Protein-quantitative trait loci (pQTLs) for the genetic variants associated with unclassified HF, HFrEF and HFpEF.

File Name: Supplementary Data 16
Description: Genome-wide significant loci overlapping with human enhancers for unclassified HF, HFrEF and HFpEF.

File Name: Supplementary Data 17
Description: Statistically significant ($p<5E-7$) associations of genetically predicted gene expression with HFrEF in 48 tissues and eQTL colocalization.

File Name: Supplementary Data 18
Description: Gene-set analysis using DEPICT for HFrEF and HFpEF.

File Name: Supplementary Data 19
Description: Pathway enrichment analysis using DEPICT for HFrEF and HFpEF.
