# Supplementary material for: Genetic architecture of heart failure with preserved versus reduced ejection fraction
Source: Nat Commun. 2022 Dec 14;13:7753. doi: 10.1038/s41467-022-35323-0 (PMC9751124; doi:10.1038/s41467-022-35323-0)
Supplement: Supplementary file 5 — Reporting Summary [file 41467_2022_35323_MOESM5_ESM.pdf]

Corresponding author(s): Jacob Joseph, Yan V. Sun

Last updated by author(s): Sep 2, 2022

Reporting Summary

Nature Portfolio wishes to improve the reproducibility of the work that we publish. This form provides structure for consistency and transparency in reporting. For further information on Nature Portfolio policies, see our Editorial Policies and the Editorial Policy Checklist.

Statistics

For all statistical analyses, confirm that the following items are present in the figure legend, table legend, main text, or Methods section.

n/a	Confirmed	
☐	☒	The exact sample size (n) for each experimental group/condition, given as a discrete number and unit of measurement
☐	☒	A statement on whether measurements were taken from distinct samples or whether the same sample was measured repeatedly
☐	☒	The statistical test(s) used AND whether they are one- or two-sided<br>*Only common tests should be described solely by name; describe more complex techniques in the Methods section.*
☐	☒	A description of all covariates tested
☐	☒	A description of any assumptions or corrections, such as tests of normality and adjustment for multiple comparisons
☐	☒	A full description of the statistical parameters including central tendency (e.g. means) or other basic estimates (e.g. regression coefficient) AND variation (e.g. standard deviation) or associated estimates of uncertainty (e.g. confidence intervals)
☐	☒	For null hypothesis testing, the test statistic (e.g. F, t, r) with confidence intervals, effect sizes, degrees of freedom and P value noted<br>*Give P values as exact values whenever suitable.*
☒	☐	For Bayesian analysis, information on the choice of priors and Markov chain Monte Carlo settings
☒	☐	For hierarchical and complex designs, identification of the appropriate level for tests and full reporting of outcomes
☐	☒	Estimates of effect sizes (e.g. Cohen's d, Pearson's r), indicating how they were calculated

Our web collection on statistics for biologists contains articles on many of the points above.

Software and code

Policy information about availability of computer code

Data collection: *Provide a description of all commercial, open source and custom code used to collect the data in this study, specifying the version used OR state that no software was used.*

Data analysis:
R statistical software, https://www.r-project.org/
PLINK, https://zzz.bwh.harvard.edu/plink/
FUMA, https://fuma.ctglab.nl/
LDSC, https://github.com/bulik/ldsc
GCTA, https://yanglab.westlake.edu.cn/software/gcta/#Overview
METAL, http://csg.sph.umich.edu/abecasis/metal/
DEPICT, https://depict.broadinstitute.org/mpg/depict
S-PrediXcan/MetaXcan, https://github.com/hakyimlab/MetaXcan
GTEx, https://www.gtexportal.org/home/

For manuscripts utilizing custom algorithms or software that are central to the research but not yet described in published literature, software must be made available to editors and reviewers. We strongly encourage code deposition in a community repository (e.g. GitHub). See the Nature Portfolio guidelines for submitting code & software for further information.

Data

Policy information about availability of data

All manuscripts must include a data availability statement. This statement should provide the following information, where applicable:

- Accession codes, unique identifiers, or web links for publicly available datasets
- A description of any restrictions on data availability
- For clinical datasets or third party data, please ensure that the statement adheres to our policy

The full summary level association data genome-wide association analyses in the MVP and the meta-analysis from this report will be available through dbGaP (accession number phs001672) at the time of publication.

Field-specific reporting

Please select the one below that is the best fit for your research. If you are not sure, read the appropriate sections before making your selection.

☒ Life sciences ☐ Behavioural & social sciences ☐ Ecological, evolutionary & environmental sciences

For a reference copy of the document with all sections, see nature.com/documents/nr-reporting-summary-flat.pdf

Life sciences study design

All studies must disclose on these points even when the disclosure is negative.

Sample size	43,344 heart failure cases (19,495 HFrEF and 19,598 HFpEF cases) and 258,943 controls from the Million Veteran Program non-Hispanic Whites; 8,227 heart failure cases and 379,788 controls from the UK Biobank cohort with European ancestry.
Data exclusions	Data were excluded if they did not pass our pre-established quality control metrics, or if they did not fall within the main ancestry groups used for analysis. Individuals were excluded if they don't meet the case or control definition of heart failure.
Replication	The GWAS associations with all-cause heart failure were replicated in the HERMES heart failure GWAS.
Randomization	Randomization is not applicable, as this is a population-based observational study of prevalent disease cases and controls.
Blinding	Blinding is not applicable, as this is a population-based observational study of prevalent disease cases and controls.

Reporting for specific materials, systems and methods

We require information from authors about some types of materials, experimental systems and methods used in many studies. Here, indicate whether each material, system or method listed is relevant to your study. If you are not sure if a list item applies to your research, read the appropriate section before selecting a response.

Materials & experimental systems

n/a	Involved in the study	
☒	☐	Antibodies
☒	☐	Eukaryotic cell lines
☒	☐	Palaeontology and archaeology
☒	☐	Animals and other organisms
☐	☒	Human research participants
☒	☐	Clinical data
☒	☐	Dual use research of concern

Methods

n/a	Involved in the study	
☒	☐	ChIP-seq
☒	☐	Flow cytometry
☒	☐	MRI-based neuroimaging

Human research participants

Policy information about studies involving human research participants

Population characteristics	MVP non-Hispanic European American participants are comprised predominantly of male subjects (92%). The average age at study enrollment ranged from 63 for controls to 70 for cases. Average body mass index (BMI) was lower in controls (29) than that in heart failure cases (31). The prevalence of heart failure risk factors including Atrial fibrillation, coronary artery disease, chronic kidney disease, diabetes, hyperlipidemia, hypertension, peripheral vascular disease and stroke were all higher in cases than that in controls (Table 1).
Recruitment	Male and female individuals older than 18 years have been recruited voluntarily from more than 60 VA Medical Centers nationwide for participation in the Million Veteran Program biobank study. Recruitment is currently occurring in person at

selected sites in the VHA health care system. Every Veteran is assigned a de-identified study ID number, which is used to track them throughout the entire process of recruitment, enrollment, sample collection and use; this approach also provides a level of protection for personal identifiers from the outset. Given that study enrollment is voluntary, biases of this study are similar to those of any mega-biobank with voluntary enrollment, including survivorship bias.

Ethics oversight	The Million Veteran Program received ethical and study protocol approval from the VA Central Institutional Review Board (IRB) in accordance with the principles outlined in the Declaration of Helsinki.

Note that full information on the approval of the study protocol must also be provided in the manuscript.
